# Robustness of Controllability for Networks Based on Edge-Attack

**DOI:** 10.1371/journal.pone.0089066

**Published:** 2014-02-26

**Authors:** Sen Nie, Xuwen Wang, Haifeng Zhang, Qilang Li, Binghong Wang

**Affiliations:** 1 Department of Modern Physics, University of Science and Technology of China, Hefei, P. R. China; 2 School of Mathematical Science, Anhui University, Hefei, P. R. China; 3 Department of Mathematics and Physics, Anhui Jianzhu University, Hefei, P. R. China; 4 College of Physics and Electronic Information Engineering, Wenzhou University, Wenzhou Zhejiang, P. R. China; 5 School of Science, Southwest University of Science and Technology, Mianyang, Sichuan, P. R. China; Wake Forest School of Medicine, United States of America

## Abstract

We study the controllability of networks in the process of cascading failures under two different attacking strategies, random and intentional attack, respectively. For the highest-load edge attack, it is found that the controllability of Erdős-Rényi network, that with moderate average degree, is less robust, whereas the Scale-free network with moderate power-law exponent shows strong robustness of controllability under the same attack strategy. The vulnerability of controllability under random and intentional attacks behave differently with the increasing of removal fraction, especially, we find that the robustness of control has important role in cascades for large removal fraction. The simulation results show that for Scale-free networks with various power-law exponents, the network has larger scale of cascades do not mean that there will be more increments of driver nodes. Meanwhile, the number of driver nodes in cascading failures is also related to the edges amount in strongly connected components.

## Introduction

Many natural and manmade systems can be modeled as a network structure which is consisted by the nodes and links [1], [2]. In the view of complex networks, the individuals in systems are presented by nodes, while the edges among nodes symbol the specific relations, such as they can sensor and transfer information between each other. For past decades, the dynamics on complex networks have been studied to understand the underlying mechanisms of systems [3]–[8]. With the deep understanding of basic principle, recently, people focus on the ultimate goal which is to develop the capability to control the systems. The dynamical system is controllable if it can be driven from any initial state to any desired final state with external inputs in finite time[9]–[11]. Though most of real systems are nonlinear systems, however, the controllability of nonlinear systems is in many aspects structurally similar to that of linear systems[12]–[15]. Liu et al.[9] studied the controllability of various real systems and proposed a method to find the minimal driver nodes which could control the whole network, the main conclusion is that the controllability of networks is determined by the degree distributions of networks. To further study this problem, Wang et al.[16] give a solution of exact controllability to find the minimum set of driver nodes required to fully control the networks with arbitrary structures and link-weights.

Generally, the systems are always confronting with the random or intentional edges attacks, for example, in power grids networks [17], the edges attacks can be interpreted as that the connections of substations are cut off so the power cannot be transmitted from one substation to others. For Internet networks, the attack on edges can be interpreted as the two Gnutella hosts cannot share Gnutella peer-to-peer file. These damages usually depend on the importance of attacked edges[18]–[20]. There are many measurements to capture the prominence of a node or an edge in the network, the betweeness centrality is a frequently used one [21]. To study the invulnerability of networks, the load of an edge or a node is conveniently defined as the betweeness centrality [18], [22]. Because of the close connections among nodes, the breakdown of nodes or edges will lead to the redistribution of physical flows or loads over other nodes or edges, then some nodes or edges break down once they are overloaded. This process is repeated until there are no overloaded nodes or edges. These cascades usually cause great losses in Internet [23], power grid [17], transportation networks [24]and so on. Sometimes, the destruction of few individuals could affect the normal daily life and result in chaos in the society, so the methods to study and reduce the damage of cascades have been proposed [25]–[27]. In Recent years, the robustness of interdependent networks has been also studied [23], [28], [29], in these networks, each node in one network depends on one node in other network, due to the existence of dependence links, the percolation transition in interdependent networks is the first-order transition which is different from the single network and the robustness of these interdependent networks under targeted attack is also different from that in single network [30].

For some networks which are easier to be controlled may be more robust to the attacks, whereas for others are opposite. Such as heterogeneous networks are harder to control and they are less robust to the intentional attacks [9], [31], because a large-scale cascading failure may be triggered by disabling a single key edge such as the highest-load one. However, from the aspect of control, the edges in network can be divided into three categories: critical, ordinary, and redundant [9], so the change of controllability under different attacking strategies will be influenced by the robustness of control [32]–[34].

In this paper, we analyze the evolution of controllability for networks in the process of cascading failures with the specific edge attacks, we find that the cascading failures are more likely to happen in the strongly connected components(SCC). Then we study the evolution of the controllability of networks with the increasing of removal fraction under two different edge attacks, random and intentional, respectively. The results show that the robustness of controllability behave differently with the increasing of removal fraction.

The paper is organized as follows: In section II, we describe the background of network controllability and the model of cascading failure. Section III are the numerical results and analysis of the networks controllability in cascading failure. Section IV is the conclusion.

## Model

### Structural controllability

For a linear, time-invariant dynamics, without the consideration of intrinsic dynamic, the node's state is governed by the following equation:
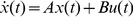
(1)where the vector 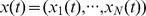 is the state of a system of 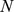 nodes at time 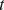, the adjacency matrix 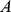 denotes the interaction strength between nodes. 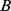 is input matrix, which defines how the input signals are connected to the nodes of networks. The system (1) is also denoted as 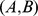, and the system is controllable if and only if controllability matrix



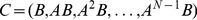
(2)has full rank. This criteria is called Kalmans controllability rank condition [35]. The 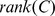 provides the dimension of the controllable subspace of the system 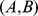. In order to fully control the network, we should choose right 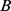 and 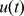 to make the matrix 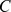 has full rank. For most real networks, it is hard to get weight of each link in adjacency matrix 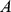, and the computation is also a prohibitive task for large networks. Therefore, the structural controllability is suitable to solve the problem, which is to choose nonzero weights in 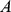 and 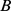 to satisfy the full rank of 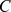, and the system can be shown to be controllable for almost all weight combinations, except for some pathological cases [9].

The exact controllability of network is determined by the maximum multiplicity of eigenvalue [16], however, the specific link-weights are not known totally in fact. Considering the computational efficiency, we choose the method of structural controllability based on the maximum matching in Ref [9]. The comparison between exact controllability and structural controllability are presented in File S1.

The maximum matching includes all edges, and none of them shares a common starting or ending node. A node is matched if an inbound edge from the maximum matching points to it, otherwise it is unmatched. The number of nodes without inbound edges from the maximum matching in network is equal to the number of input signals required for structural controllability. The system is fully controlled over a directed network while we directly control each unmatched node and there are directed paths from the input signals to all matched nodes, we call the unmatched nodes are driver nodes. Though the patterns of maximum matching may be various, the number of unmatched nodes is the same in every matching. The number of driver nodes 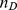 can be used to measure the controllability of network and it could be calculated by the maximum matching algorithm [9].

Meanwhile, according to the different roles in controllability of networks, the edges can be clarified into three categories [9]: ‘critical’, its removal needs to increase the number of driver nodes to maintain fully control; ‘redundant’, its removal cannot affect the current set of driver nodes; ‘ordinary’, if it is neither critical nor redundant.

### The cascading dynamics

We suppose that each edge 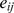 is assigned with a capacity according to its load. The load on edge 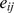 is the total number of shortest paths in network passing through the 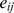 at time 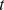. The capacity of an edge is the maximum load that the edge can handle. We assume the capacity 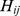 of edge 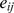 is proportional to its initial load 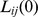 [18]:
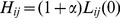
(3)where 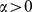 is a tolerant parameter. As we remove one or some edges in network, the distribution of shortest paths will be changed and the loads on some edges may increase and become larger than their capacity, then the overloaded edges fail and result in a new distribution of loads on edges. Finally, the cascading failure will stop as there are no overloaded edges after a few steps. Note that the loads on edges in cascades are different from the link weights of adjacency matrix in structural controllability theory.

**Figure 1:**
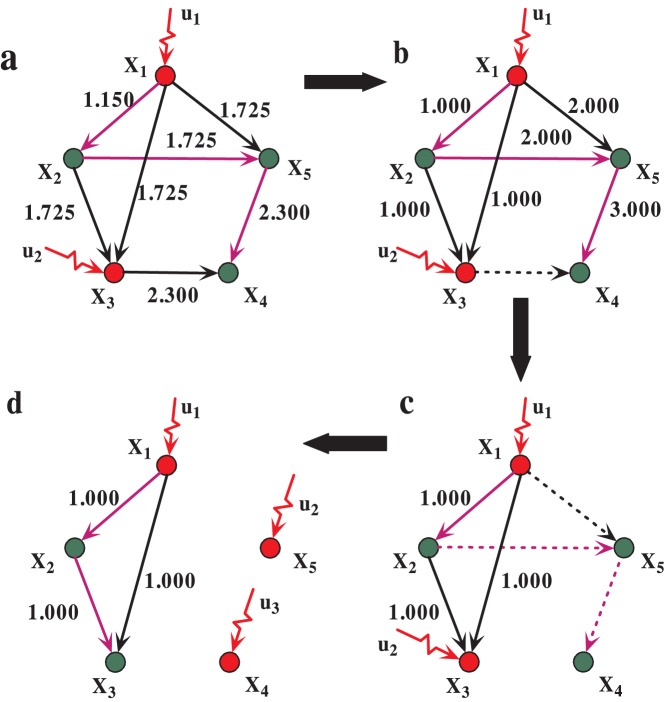
Demonstration of the controllability of network in cascading failure. The network with size 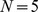 and 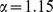, the green nodes are matched nodes and the red ones are unmatched. The purple edges are matching edges in maximum matching. However, the unmatched nodes 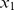, 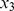 are the driver nodes which should be controlled directly by the inputs 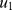 and 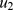, and after the edges failure, the unmatched nodes 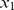, 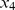, 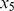 should be controlled by inputs 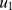, 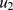 and 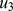. The red and green circles symbol the unmatched nodes and matched nodes, respectively. The purple lines symbol edges in maximum matching, and the dash lines symbol the failed edges in cascades. The red arrows symbol the input signals. The process of a,b,c,d shows the change of controllability in cascading failure.

We consider two different attacking strategies: (1) random attack (RA): a fraction of edges is randomly removed. (2) intentional attack (IA): a fraction of edges is removed in descending order of the initial loads. As demonstrated in Fig. 1, the network of a given degree distribution with size of 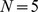, as the highest load edge 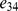 is removed, the loads of edges 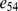, 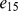, 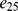 become larger than the capacities which they could handle, so they fail and be removed, then this process will be repeated until the loads of all edges are less than their capacities. Because of the change of topological structure, the pattern of maximum matching changes and the number of driver nodes grows up.

## Analysis

**Figure 2:**
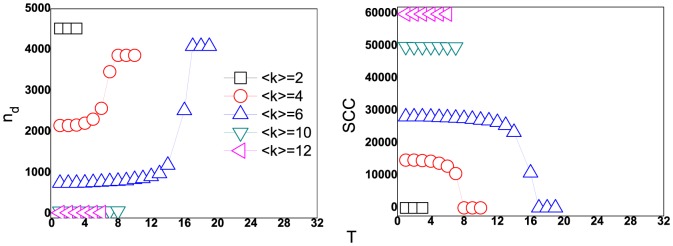
The number of driver nodes and size of SCC for ER networks with different average degrees when the highest load edge is removed. (a) the number of driver nodes at different stages of the cascading failure; (b) the amount of edges in SCC at different stages of the cascading of failure. The networks size 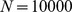 and tolerant parameter 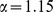. The squares, circles, triangles, lower triangles and left triangles symbol the controllability of ER networks with the average degrees 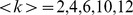 respectively.

Firstly, we investigate the controllability of directed ER networks at different stages of the cascading failures, as triggered by removal of the highest load edge. As shown in Fig. 2(a), for small average degree, the number of the shortest paths through an edge is small, and the removal of the highest load edge produces some unreachable pairs nodes, so the loads of edges which were on the former paths between these pairs nodes decrease and the edges will not overload. In this case, there are only a small fraction of failed edges, and these edges may belong to ordinary and redundant edges, so the increment of driver nodes is small.

For the moderate average degrees, the networks need more iteration steps to reach the stable state with the increasing of average degree, which indicates more edges are failed and the networks need more driver nodes to achieve fully control. As the highest load edge is removed, most of nodes can also reach each other, however, the loads of edges on the alternative shortest paths usually exceed their capacities, the redistribution of loads makes cascade can spread in networks. It can be seen in Fig. 2(a), at the beginning of cascade, the network with average degree 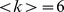 needs few driver nodes, but finally, the cascading failure makes it to need more driver nodes. While, for large average degrees, the enough alternative paths cannot change the balance of loads in cascading failures and hardly leads to a global redistribution of loads over all the network, then, there are few overloaded edges and the controllability of networks changes slightly.

The size of the largest connected component is usually used to quantify the damage caused by a cascade, and it may relate to the controllability of network, so we study the number of edges in strongly connected component of the network in cascading failure. As shown in Fig. 2(b), the number of driver nodes in the process of cascading relates to the number of edges in SCC, especially at the critical stage of cascade, in which the largest scale of cascades occurs and the increment of driver nodes is maximum.

**Figure 3:**
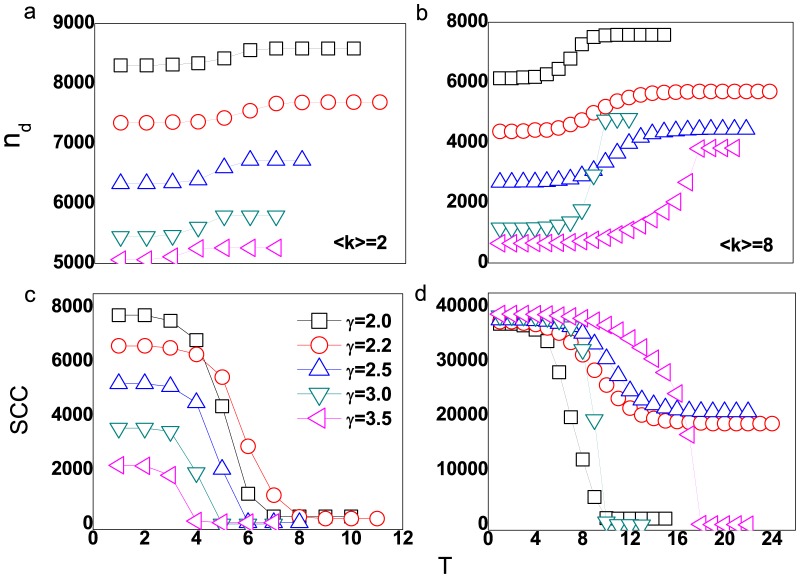
The number of driver nodes and size of SCC for SF networks with different average degrees and power-law exponents when the highest load edge is removed. (a) the number of driver nodes at different stages of cascading failure with average degree 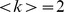; (b) 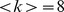; (c) the amount of edges in SCC at different stages of cascading failure with average degree 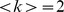; (d) 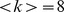. The networks size 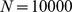 and tolerant parameter 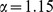. The squares, circles, triangles, lower triangles and left triangles symbol the controllability of SF networks with the 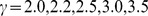 respectively.

Compared with ER networks, we can find that the cascading failure also occur even for small average degree in SF networks as shown in Fig. 3(a) and Fig. 3(c). The network exists the strongly connected component even for small average degree because of the existence of hub nodes, then the cascades can be triggered by the removal of the highest load edge. In addition, the damage scale for the networks with small power-law exponents are larger than that with large power-law exponents. However, the increments of driver nodes for both of these networks are nearly the same. The number of driver nodes for SF network with average degree 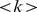 and power-law exponent 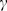 is given by:
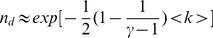
(4)


The number of driver nodes after the cascades is approximatively given by:
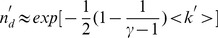
(5)where we assume the degree distribution has not been changed after cascading failure, and the average degree after cascades is 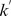, then the increments of driver nodes 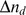 for small 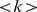 and 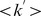 is approximatively:
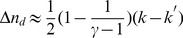
(6)


The Eq. 6 depicts that even the networks with small power-law exponents after large scale cascades, the increment of driver nodes are the same as those with large power-law exponents. On the other hand, for large average degree as shown in Fig. 3(b) and Fig. 3(d), the cascading failure spreads widely in network, which leads to the more driver nodes. Except for the case of 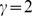, the increments of driver nodes for the networks with large power-law exponents are larger than that with small ones, that is because there are more failed edges in cascades for large power-law exponents networks.

**Figure 4:**
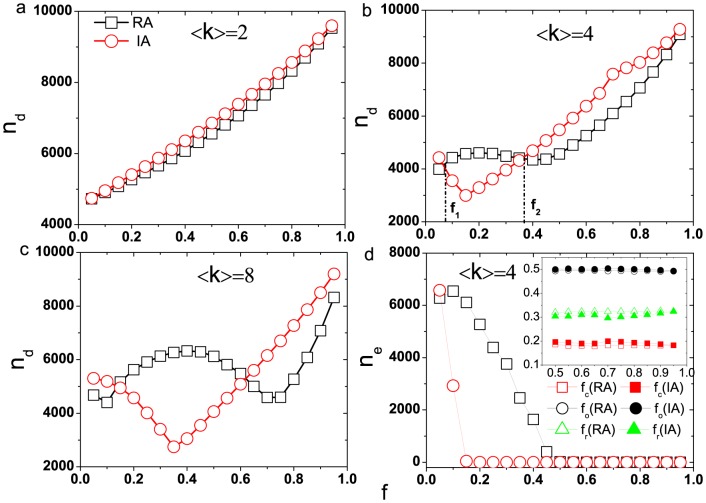
The number of driver nodes as a function of removal fraction 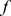 for ER networks under different attacks. (a) 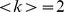, (b) 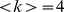, (c) 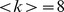, (d) the amount of failed edges in cascades as a function of removal fraction 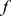 for 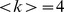. The inset of (d) shows the densities of three categories edges in all failed edges with the increasing of 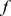 for 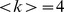. The networks size 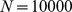 and tolerant parameter 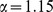. Each data point result is obtained by averaging over 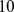 different realizations. The squares symbol the random attack and the circles symbol the intentional attack. In the inset of Fig. 4(d), the squares, circles and triangles symbol the critical, ordinary and redundant edges respectively. The open symbols and solid symbols represent the amount of edges under random and intentional attack respectively.

Next, we discuss the attacks on the networks which are represented by the removal of a fraction of edges in networks. In Fig. 4, we present the results of the number of driver nodes as a function of removal fraction 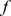 in ER networks. We can find that for the small average degree, both the random and intentional attacks cannot cause the cascading failure in ER network which is agreement with the results in Fig. 2, thus the influence of both attacking strategies on the number of driver nodes are nearly the same. However, for larger average degrees, the curves of the number of driver nodes under the two attacking strategies can be divided into three regions as presented in Fig. 4(b) and (c). In the following, we take the Fig. 4(b) as an example to illustrate:

(i) 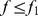

In this region, the amount of failed edges triggered by intentional attack is larger than that by random attack, this is similar with the highest-load edge attack situation. Thus, the network under intentional attack needs more driver nodes to achieve fully control after cascades.

(ii) 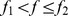

For moderate 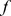, the strategy of random attack causes the network to need more driver nodes to fully control the ER networks than intentional attack strategy. With the increasing of removal fraction 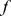, the intentional attack strategy removes a fraction of highest loads edges and this reduces the connectivity of networks, then the loads of the remaining edges which were on the former connected paths will decrease and they are hard to overload. On the contrary, the random attack strategy changes the connectivity slightly.

The total number of failed edges in cascades as a function of removal fraction 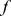 under two attacking strategies are shown in Fig. 4(d), and the critical value 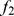 is corresponding to the removal fraction when no edges failed by cascades under both attacking strategies. We can find that the cascade which is triggered by random attack causes much more edges to fail than that by intentional attack, so in this region, the number of driver nodes needed to control network under random attack is larger than that under intentional attack.

(iii) 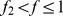

As 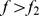, there will be no failed edges which are caused by cascades for both attacking strategies except the original removed ones. However, if we remove the same fraction of edges, the Fig. 4(b) shows that the network needs more driver nodes under intentional attack than the random attack. This result is determined by the robustness of control.

In the inset of Fig. 4(d), we present the densities of three categories edges in all failed edges with the increasing of removal fraction 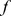. The densities of ordinary edges under both attacking strategies are nearly the same, while the random attack has larger probability to remove the redundant edges, on the contrary, the intentional attack has larger probability to remove the critical edges. This suggests that the highest loads edges in ER networks are more likely to be critical edges. Hence, in this region, the number of driver nodes under intentional attack is larger than that under random attack. For ER networks, there exist some directed tree-like structures, the edges on the stems always have large loads, from the aspect of control, these edges usually belong to critical. On the contrary, the edges attached to the stems belong to redundant. The random attack has large probability to remove the redundant edges, but their removal affects the controllability slightly, so the network needs less driver nodes to fully control under random attack in this region.

**Figure 5:**
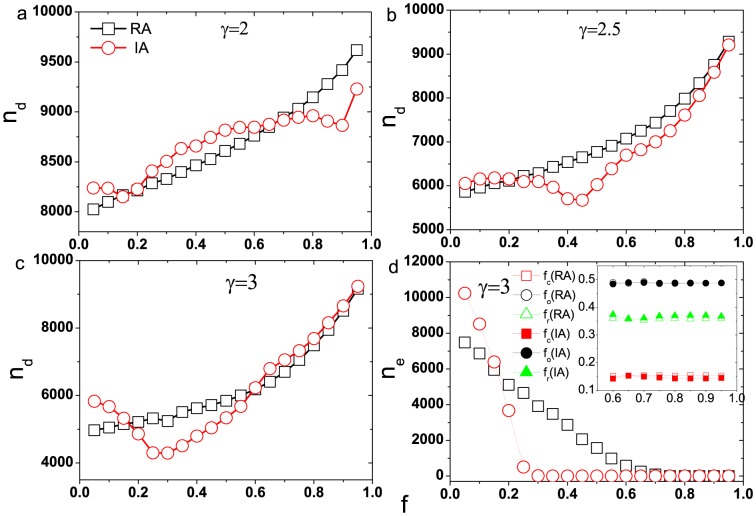
The number of driver nodes as a function of removal fraction 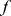 for SF network under different attacks. (a) 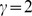, (b) 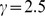, (c) 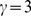, (d) the amount of failed edges in cascades as a function of removal fraction 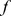 for 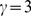. The inset of (d) shows the densities of three categories edges in all failed edges with the increasing of 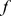 for 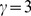. The networks size 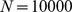, 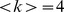 and tolerant parameter 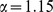. Each data point result is obtained by averaging over 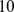 different realizations. The squares symbol the random attack and the circles symbol the intentional attack. In the inset of Fig. 5(d), the squares, circles and triangles symbol the critical, ordinary and redundant edges respectively. The open symbols and solid symbols represent the amount of edges under random and intentional attack respectively.

The results for SF networks are demonstrated in Fig. 5(a)-(d), which have many differences comparing with the ER networks: (i) In the region of moderate 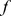, the intentional attack causes the network need more driver nodes than random attack as shown in Fig. 5(a), this is different from the case of ER networks and the cases of other two SF networks which are shown in Fig. 5(b) and Fig. 5(c). The SF network with power-law exponent 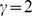 is more heterogenic, in such network, there exists a lot of hub nodes and the edges among these nodes are quite dense, so the pairs of nodes can also be reached through these hub nodes even when we remove a fraction of edges with highest loads, but the remaining edges of hub nodes are easier to overload and fail. This leads to a larger damage in network under intentional attack than random attack; (ii) In the region of larger 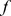, the networks with small power-law exponent 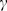 need more driver nodes to maintain fully control under random attack than intentional attack. For larger 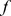, the intentional attack trends to remove the ordinary edges, and this removal sometimes cannot increase the number of driver nodes, therefore, the result is opposite to that in ER networks; (iii) For larger 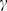, the difference of number of driver nodes under two attacking strategies is not significant as larger 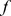. It can be seen from the inset of Fig. 5(d). The densities of three categories edges in all failed edges are nearly the same, thus the number of the driver nodes under both attacking strategies are nearly the same too.

**Figure 6:**
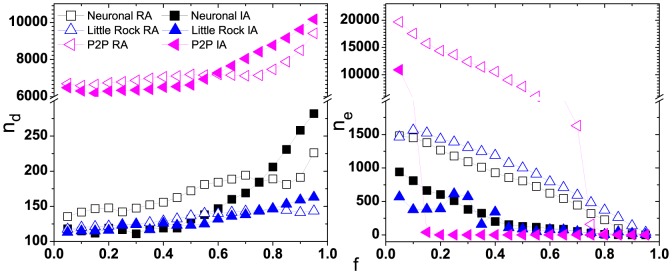
The results of cascading failure for several real networks: Internet p2p network, Neural network of Caenorhbditis elegans and Food web in Little Rock lake. (a) The number of driver nodes as a function of removal fraction 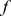 under two attack strategies, (b) The amount of failed edges in cascades as a fraction of 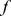. The squares, triangles and left triangles symbol the Neural network of Caenorhbditis elegans, the Food web in Little Rock lake and the Internet p2p network respectively. The open symbols and solid symbols represent the controllability of networks under random and intentional attack respectively.

Finally, we simulate the two attacking strategies on the real systems, Internet peer-to-peer network [36], Neural network of Caenorhbditis elegans [1] and Food web in Little Rock lake [37]. As demonstrated in Fig. 6, with the increasing of removal fraction 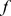, the change of controllability and the amount of failed edges show the same tendency as that in SF networks.

To further study the controllability of networks with random weights and identical weights, we present the number of driver nodes calculated by exact controllability and maximum matching algorithm for several networks in File S1. For the case of identical weights, the number of driver nodes in ER networks and SF networks for two methods are the same; however, in the real systems, the number of driver nodes obtained by the exact controllability is always larger than that by structural controllability in the process of cascading. For the case of random weights, all of networks show no difference between two methods.

## Discussion

In conclusion, we study the controllability of networks in cascading failure under two different attacking strategies, random and intentional, respectively. As we remove the highest load edge in network, the number of driver nodes in cascading failure mainly depends on the edges amount in strongly connected component(SCC), the reason is that the cascading failure is more likely to spread in SCC. For ER networks, with small or large enough average degrees, the highest-load attack cannot trigger the cascades and it impacts the controllability of networks slightly. The existence of hub nodes makes the SF networks are vulnerable to the highest-load attack, and the larger damage can be triggered in the network with small power-law exponent than with the large one when the average degree is small, however, the increments of the driver nodes for both of them are nearly the same.

For multiple edges attacks represented by removing a fraction 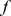 of edges. The moderate 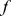 can efficiently suppresses the cascade under intentional attack, which causes the number of driver nodes under intentional attack is smaller than the case under random attack for ER networks and SF networks with moderate 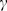. This result indicates that the random attack impacts the controllability of less heterogenic networks more greatly than intentional attack for moderate removal fraction 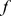. For larger 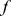, the attacks of both two strategies cannot trigger the cascades, and the number of driver nodes is determined by the robustness of control.

## Supporting Information

File S1
**This file includes supporting materials and Figure S1–S4.** Figure S1, the blue squares, green circles, red lower triangles, light blue triangles, and purple diamonds symbol the structural controllability of networks with average degree 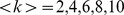 respectively. The blue, green, red, light blue and purple dash lines symbol the exact controllability of networks with average degree 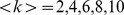 respectively. Figure S2, the blue lower triangles, green triangles, red diamonds, light blue circles and purple squares symbol the structural controllability of networks with 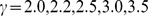 respectively. The blue, green, red, light blue and purple dash lines symbol the exact controllability of networks with 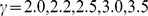 respectively. Figure S3, the diamonds, lower triangles, circles and squares symbol the Little Rock, Ythan, Grassland and Seagrass networks respectively. The open symbols and solid symbols represent the exact and structural controllability of networks respectively. Figure S4, the diamonds, lower triangles, circles and squares symbol the Little Rock, Ythan, Grassland and Seagrass networks respectively. The open symbols and solid symbols represent the exact and structural controllability of networks respectively.(ZIP)
