# Supplementary material for: Robustness of Controllability for Networks Based on Edge-Attack
Source: PLoS One. 2014 Feb 26;9(2):e89066. doi: 10.1371/journal.pone.0089066 (PMC3935847; doi:10.1371/journal.pone.0089066)
Supplement: File S1 — This file includes supporting materials and Figure S1–S4. Figure S1, the blue squares, green circles, red lower triangles, light blue triangles, and purple diamonds symbol the structural controllability of networks with average degree respectively. The blue, green, red, light blue and purple dash lines symbol the exact controllability of networks with average degree respectively. Figure S2, the blue lower triangles, green triangles, red diamonds, light blue circles and purple squares symbol the structural controllability of networks with respectively. The blue, green, red, light blue and purple dash lines symbol the exact controllability of networks with respectively. Figure S3, the diamonds, lower triangles, circles and squares symbol the Little Rock, Ythan, Grassland and Seagrass networks respectively. The open symbols and solid symbols represent the exact and structural controllability of networks respectively. Figure S4, the diamonds, lower triangles, circles and squares symbol the Little Rock, Ythan, Grassland and Seagrass networks respectively. The open symbols and solid symbols represent the exact and structural controllability of networks respectively. (ZIP) [file pone.0089066.s001.zip › File S1/SupportingInformation.pdf]

Figure S1. (Color online) Comparison between the exact controllability and structural controllability in ER networks. The symbols denote the number of driver nodes calculated by maximum matching algorithm, the lines denote the number of driver nodes of exact controllability. The network size $N = 1000$.

Figure S2. (Color online) Comparison between the exact controllability and structural controllability in SF networks. The symbols denote the number of driver nodes calculated by maximum matching algorithm, the lines denote the number of driver nodes of exact controllability. The network size $N = 1000$, $< k >= 2$.

Supporting Information: Comparison between exact controllability and structural controllability

Considering the number of driver nodes of structural controllability calculated by maximum matching algorithm is agreement with the result of exact controllability when the link weights are chosen randomly. For the case of identical weights, there exist differences between two of methods. Hence, it is necessary for us to study whether the results obtained by the structural controllability are the same as the exact controllability. In the following, we present the results of structural controllability and exact controllability for directed ER networks, directed Scale-free networks and several real networks with identical weights and random weights, respectively. The attack strategy we used in the following is represented by removing the highest-load edge.

The main conclusions of the following simulations are:

(i) For ER networks and Scale-free networks with identical weights($\omega_{ij} = 1$), the numbers of driver nodes calculated by two methods are the same. This is caused by the effect of weights to the controllability is insignificant, so the results for both of them are same as shown in Figure S1 and Figure S2.

(ii) For several real networks (Food web) with identical weights, the numbers of driver nodes calculated by two methods are different. The number of driver nodes of exact controllability is always larger than the case of structural controllability as shown in Figure S3. For the case of random weights, the results for exact controllability and structural controllability are same as shown in Figure S4.

1. ER networks with identical link weights ω_{ij}

2. SF networks with identical link weights ω_{ij}

3. Real networks

3.1 Food web networks with identical link weights $\omega_{ij} = 1$

3.2 Food web networks with random weights

Figure S3. (Color online) Comparison between the exact controllability and structural controllability for food web networks with identical weights. The filled symbols denote the number of driver nodes calculated by maximum matching algorithm, the open symbols denote the number of driver nodes of exact controllability.

Figure S4. (Color online) Comparison between the exact controllability and structural controllability for food web networks with random weights. The symbols denote the number of driver nodes calculated by maximum matching algorithm, the lines denote the number of driver nodes of exact controllability.
